# Danger of Scopolamin-Morphin Anesthesia

**Published:** 1907-03

**Authors:** 


### DANGER OF SCOPOLAMIN-MORPHIN ANESTHESIA.

---

Wood selected from the literature 1,988 cases of Scopolamin-morphin anesthesia. In every instance he consulted the original authorties (except one). In this series there were 23 deaths, of which, after careful reading of the descriptions of the symptoms and the author's own conclusions, Wood thinks it justifiable to attribute the death to the anesthetic in 9 instances. The mortality in this method reaches, therefore, one death in 221 anesthesias, as against one death in 1,500 from ether. The anesthesia was unsatisfactory in at least 868 cases, and ether or chloroform was administered during the operation. A considerable experience with both ether alone and morphin-ether anesthesia in dogs has convinced Wood that although far more convenient, the preceding use of morphin rather increases than diminishes the likelihood of anesthetic accidents. He says that there is no evidence as to the special indications for the preference of this method over ether, and that certainly it is not in cases of advanced renal disease, for every clinician will hesitate seriously before giving one-half of a grain of opium to a nephritic. In view of the facts that the combination of hysocin and morphin for the production of surgical anesthesia is scientifically irrational, and has yielded a mortality of over 4 per 1,000, and that in 69 per cent. of the cases the anesthesia was unsatisfactory, Wood concludes that it must be either a very bold or a very ignorant surgeon who will persist in its use.—Amer. Med., December, 1906.

---

A hasty diagnosis of ulcer in the stomach should not be made merely because the patient has vomited suddenly large quantities of blood. If the bleeding occurs at regular intervals the possibility of vicarious menstruation must be considered.—American Journal of Surgery.

---

### COMMENDATIONS OF WOLFF'S DERMATOLOGY.

"It is concise, and all I could say would be in praise of it."---*Dr. H. A. Robbins, Washington, D. C.*

"An interesting contribution to the subject of which it treats.' "---*American Journal of Dermatology.*

"In this work Dr. Wolff has undertaken the difficulttask of covering the subject of dermatology in 275 pages and it may be said at once that he has succeeded better than might have been expected in so difficult an undertaking. He writes in a smooth, running style, which, while condensed, is clear and agreeable. While most of the diseases are considered briefly the salient facts are always given and treatment is granted sufficient consideration. The work isconservative, but up to date and can be fully recommended. The publisher has done his part well."--*Clinical Review.*

Cleveland Press, Ogden Ave. aud Lincoln St., Chicago, or Lester Book & Stationary Co., Atlanta, Ga.

---

Attention is called to the fact that all the salicylic acid in the Tongaline Preparations is made by the Mellier Drug Company from natural sources.

